# Construction and Validation of a 13-Gene Signature for Prognosis Prediction in Medulloblastoma

**DOI:** 10.3389/fgene.2020.00429

**Published:** 2020-05-19

**Authors:** Chang Li, Han Zou, Zujian Xiong, Yi Xiong, Danielle F. Miyagishima, Siyi Wanggou, Xuejun Li

**Affiliations:** ^1^Department of Neurosurgery, Xiangya Hospital, Central South University, Changsha, China; ^2^Xiangya School of Medicine, Central South University, Changsha, China; ^3^Hunan International Scientific and Technological Cooperation Base of Brain Tumor Research, Xiangya Hospital, Central South University, Changsha, China; ^4^Department of Neurosurgery, Yale School of Medicine, New Haven, CT, United States; ^5^Department of Genetics, Yale School of Medicine, New Haven, CT, United States

**Keywords:** medulloblastoma, gene signature, prognosis, risk score model, LASSO

## Abstract

**Background:** Recent studies have identified several molecular subgroups of medulloblastoma associated with distinct clinical outcomes; however, no robust gene signature has been established for prognosis prediction. Our objective was to construct a robust gene signature-based model to predict the prognosis of patients with medulloblastoma.

**Methods:** Expression data of medulloblastomas were acquired from the Gene Expression Omnibus (GSE85217, *n* = 763; GSE37418, *n* = 76). To identify genes associated with overall survival (OS), we performed univariate survival analysis and least absolute shrinkage and selection operator (LASSO) Cox regression. A risk score model was constructed based on selected genes and was validated using multiple datasets. Differentially expressed genes (DEGs) between the risk groups were identified. Kyoto Encyclopedia of Genes and Genomes (KEGG), Gene Ontology (GO), and protein–protein interaction (PPI) analyses were performed. Network modules and hub genes were identified using Cytoscape. Furthermore, tumor microenvironment (TME) was evaluated using ESTIMATE algorithm. Tumor-infiltrating immune cells (TIICs) were inferred using CIBERSORTx.

**Results:** A 13-gene model was constructed and validated. Patients classified as high-risk group had significantly worse OS than those as low-risk group (Training set: *p* < 0.0001; Validation set 1: *p* < 0.0001; Validation set 2: *p* = 0.00052). The area under the curve (AUC) of the receiver operating characteristic (ROC) analysis indicated a good performance in predicting 1-, 3-, and 5-year OS in all datasets. Multivariate analysis integrating clinical factors demonstrated that the risk score was an independent predictor for the OS (validation set 1: *p* = 0.001, validation set 2: *p* = 0.004). We then identified 265 DEGs between risk groups and PPI analysis predicted modules that were highly related to central nervous system and embryonic development. The risk score was significantly correlated with programmed death-ligand 1 (*PD-L1*) expression (*p* < 0.001), as well as immune score (*p* = 0.035), stromal score (*p* = 0.010), and tumor purity (*p* = 0.010) in Group 4 medulloblastomas. Correlations between the 13-gene signature and the TIICs in Sonic hedgehog and Group 4 medulloblastomas were revealed.

**Conclusion:** Our study constructed and validated a robust 13-gene signature model estimating the prognosis of medulloblastoma patients. We also revealed genes and pathways that may be related to the development and prognosis of medulloblastoma, which might provide candidate targets for future investigation.

## Introduction

Medulloblastoma is the most common central nervous system (CNS) malignancy in children (Louis et al., 2016; Northcott et al., 2019). The current revision of WHO classification of medulloblastoma integrates histological and molecular studies (Louis et al., 2016). It is established that medulloblastoma can be characterized into at least four major subgroups, Wingless (WNT), Sonic hedgehog (SHH), group 3, and group 4 (Taylor et al., 2012). While the WNT and SHH subgroup can be clearly identified based on the WNT and SHH signaling pathway mutations, much less is known about Group 3 and Group 4 tumors, and these subgroups remain as non-SHH/non-WNT medulloblastomas in WHO’s 2016 classification for diagnostic considerations (Louis et al., 2016). Group 3 and Group 4 tumors have few mutations but can be identified through transcriptional profiles (Ramaswamy et al., 2016b). Despite the difficulty in classification—and an overlap (intermediate 3/4 Group) between these tumors has been observed—Group 3 and Group 4 are indeed different subgroups featuring distinct clinicopathological and genomic characteristics (Łastowska et al., 2018; Schwalbe et al., 2019). Group 3 tumors have the worst prognosis of all medulloblastomas, whereas Group 4 tumors have an intermediate prognosis similar to that of SHH subgroup (Taylor et al., 2012). Although large cell/anaplastic (LCA) tumors can be found in all four molecular subgroups, the majority of this histological subtype are found in Group 3 tumors. Moreover, Group 3 tumors tend to have high levels of expression of the MYC proto-oncogene, bHLH transcription factor (*MYC*), whereas *MYC* expression in Group 4 tumors are relatively low. On the other hand, isochromosome 17q can be commonly seen in Group 4 tumors (approximately 66%), whereas it is less common in Group 3 tumors (approximately 26%) (Kool et al., 2012). While molecular subgroups improved our knowledge of medulloblastoma, there are still some limitations, particularly in the characterization of clinical outcomes. Wide variation in patient outcomes within the same subgroup has been observed (Ramaswamy et al., 2016b), and many subgroups show a subsequent level of structures, namely, subtypes of molecular subgroups (Taylor et al., 2012). Labeled with Greek letters, such as α, β, γ, etc., these subtypes are associated with distinct clinical outcomes. For example, study from Cho et al. (2011) demonstrated that Group 3β medulloblastomas have a clinical outcome similar to Group 4 tumors. However, the number of subtypes for each subgroup and the extent of overlap between subgroups remains unknown. Cavalli et al. (2017) identified 12 subtypes of the known molecular subgroups in their study of 763 medulloblastoma cases, while new subtypes featuring hotspot in-frame insertions that target Kelch repeat, BTB domain containing 4 (*KBTBD4*), and “enhancer hijacking” events that activate PR/SET domain 6 (*PRDM6*) were proposed in a recent study (Northcott et al., 2017). Therefore, a precise prognostic model with high efficacy and broad applicability would assist in prognostic prediction of the patients with medulloblastoma in addition to the molecular and histology characterization. In this study, we utilized expression data from the Gene Expression Omnibus (GEO) to construct a 13-gene signature that can robustly predict risk stratification of patients with specific identification of a high-risk group with significant worse overall-survival (OS) among medulloblastoma patients. We validated the efficacy and applicability of the model using two unique datasets sequenced using different platforms. Using this model, we identified differentially expressed genes (DEGs) and performed a pathway enrichment analysis. Furthermore, we performed comprehensive analyses of the protein–protein interaction (PPI), tumor microenvironment (TME), tumor-infiltrating immune cells (TIICs), and immune checkpoints of the medulloblastoma samples, and we assessed their correlation with our risk score model, to give insight into the underlying mechanism of the 13-gene signature and its relation with published molecular signatures of the disease. Our study has potential clinical significance in patient management and may shed light on the tumorigenesis of medulloblastoma.

## Materials and Methods

### Patient Cohorts and Data Preprocessing

Expression datasets of medulloblastomas (GSE85217, *n* = 763; GSE37418, *n* = 76) were acquired from GEO^1^ (Robinson et al., 2012; Morfouace et al., 2015; Cavalli et al., 2017; Ramaswamy and Taylor, 2019). Clinical data, including gender, histology, age, and molecular subgroup, were retrieved from corresponding publications (Robinson et al., 2012; Morfouace et al., 2015; Cavalli et al., 2017; Ramaswamy and Taylor, 2019). Patients without survival information were excluded. Considering the distinct clinical characteristics of infant medulloblastoma (Waszak et al., 2018), cases that were 3 years old or younger were excluded. To remove the batch effect (Luo et al., 2010), expression data were normalized using a quantile normalization method via the “limma” R package and log2 transformed (Ritchie et al., 2015). Outliers were detected using the “hclust” R package (Müllner, 2013) and excluded. Probes were mapped to genes per manufacturer’s instruction for each microarray platform when applicable (GRL22286, Affymetrix, United States^2^; GRL570, Affymetrix, United States^3^). For genes detected by multiple probe sets without recommended probes from the manufacturer, the probe with the highest expression covering the targeted region was selected for analysis. Probes without descriptions from the manufacturer were excluded. After data preprocessing, we randomly assigned cases in dataset GSE85217 to the training set (70%) or validation set 1 (30%) with proportionate stratification by the four molecular subgroups (SHH, WNT, Group 3, and Group 4). GSE37418 was assigned as validation set 2 for external validation. Of note, the datasets were sequenced on different microarray platforms (GPL22286 for GSE85217; GPL570 for GSE37418).

### Construction of the Gene Signature Model

The training set was used to construct the prognostic model. The univariate survival analysis was performed using the R packages “survival” and “surveminer” (Therneau and Grambsch, 2000) to identify OS-related genes. OS-related genes were defined as genes that were significantly associated with the OS (*p* < 0.05) in the univariate survival analysis. We then used a least absolute shrinkage and selection operator (LASSO) Cox proportional hazards model to identify signature genes predicting the OS of the patients. The optimal penalty parameter was estimated by 10-fold cross-validation in the training dataset (Tibshirani, 1997; Therneau and Grambsch, 2000). Genes with none-zero coefficients were selected for further model construction. Risk scores for each patient was calculated using the following formula:

$$\text{Risk Score}=\sum_{i=1}^{n}E\,x\,p_{i}\times L_{i}$$

n, *Exp*_*i*_, and *L*_*i*_, represented the number of signature genes, gene expression level, and the coefficient of the gene, respectively. The cutoff between high-risk and low-risk groups was statistically estimated using the “maximally selected rank statistics method” (Lausen and Schumacher, 1992) with the training set.

### Validation of the Gene Signature Model

Using the gene signature-based model constructed from the training set, risk scores were calculated for all patients in the validation set 1 and 2. Patients were then classified as being in a “high-risk” or “low-risk” group based on their risk scores, using the cutoff estimated from the training set. To validate the performance of the gene signature model, Kaplan–Meier survival (K–M) curves were plotted for “high-risk” and “low-risk” groups. The area under the curve (AUC) of the receiver operating characteristic (ROC) for 1-, 3-, and 5-year OS were calculated for risk scores.

To assess the independent predictive value of the gene signature when considering clinical factors, univariant and multivariant analyses were performed by integrating the age, gender, tumor histology, molecular subgroups, as well as risk groups of the patient. To visualize the performance of the risk score model, heatmaps were plotted using R package “ggplot2” by clustering signature genes and risk groups.

### Differentially Expressed Gene (DEGs) Analysis

DEGs between the high-risk and low-risk group were identified using R package “limma.” Genes with fold change > 1.5 or <0.5 (Dalman et al., 2012), and adjusted *p*-value < 0.01 after Benjamini–Hochberg (BH) multiple test adjustment were considered differentially expressed. The Gene Ontology (GO) enrichment analysis was performed for DEGs using R package “clusterProfiler” and R/Bioconductor annotation data package “org.Hs.eg.db” (Yu et al., 2012; Carlson, 2019). The Kyoto Encyclopedia of Genes and Genomes (KEGG) analysis was performed using WEB-based Gene SeT AnaLysis Toolkit^4^, with hypergeometric test statistical method and BH method applied. The “TOP” method was used to identify enriched categories in which the top most significant categories were selected after ranking based on the false discovery rate (FDR).

### Protein–Protein Interaction (PPI) Analysis

Protein–protein interaction network of the DEGs was constructed using The Search Tool for the Retrieval of Interacting Genes (STRING^5^) (Szklarczyk et al., 2019). The constructed network was then visualized using Cytoscape software. The Molecular Complex Detection (MCODE) plugin was used to identify significant modules of the PPI network, and modules with score ≥ 4 and nodes ≥ 20 were included in this study (Bader and Hogue, 2003). A GO enrichment analysis were performed for DEGs in each module for functional enrichment analysis. Hub genes of the PPI network were identified using CytoHubba based on the predication of two topological analysis methods, maximal clique centrality (MCC) and Degree (Chin et al., 2014).

### Analysis of Tumor Microenvironment (TME) and Estimation of Tumor Purity as Well as Stromal and Immune Cell Admixture

Tumor microenvironment plays an important role in the development and prognosis of tumors, and the main components of TME are immune and stromal cells (Koelwyn et al., 2017). To infer the stromal, immune, and other non-tumor component admixture in the TME of medulloblastomas, immune score, stromal score, and tumor purity of each sample were calculated using the Estimation of STromal and Immune cells in MAlignant Tumor tissues using an Expression data (ESTIMATE) algorithm (Yoshihara et al., 2013). We then compared the immune score, stromal score, and tumor purity between the high-risk and low-risk group classified by our risk model, and we also assessed their correlations with the risk score.

### Analysis of Tumor Infiltrating Immune Cells (TIICs)

CIBERSORTx^6^ is an algorithm developed to estimate the abundance of TIICs based on gene expression data. Using the CIBERSORTx algorithm, we profiled 22 immune cells in each medulloblastoma sample. We then analyzed the correlation between the fraction of the immune cells and the expression level of the newly identified candidate genes (including signature genes and network hub genes). We also compared the fraction of infiltrating immune cells between the high-risk and low-risk group indicated by the 13-gene signature model.

### Statistical Analysis

R software version 3.6.2 and SPSS software version 21 were used for all statistical analysis. Spearman’s correlation between continuous variables, such as risk score, level of gene expression (log2 transformed), immune score, stromal score, tumor purity, and immune cell fraction, were evaluated, and *p*-values were adjusted using the BH method. Continuous variables between different subgroups of medulloblastomas were compared using Wilcoxon rank-sum test (Mann–Whitney *U* test), or Kruskal–Wallis H test followed by Dunn’s *post hoc* tests for pairwise comparisons. The *p*-value for survival analysis was calculated using R package “survminer” as aforementioned, and pairwise comparisons were performed using “pairwise_survdiff” function. The calculated p values were adjusted using the BH method provided by the package. *P* < 0.05 was considered statistically significant.

## Results

### Characteristics of the Studied Cohorts

After data preprocessing, 531 of 763 cases in the GSE85217 dataset and 70 of 76 cases in the GSE37418 dataset were included in this study (Table 1). Cases in GSE85217 were proportionately stratified by molecular subgroups and randomly assigned as training set (371, 70%) or validation set 1 (*n* = 160, 30%). Additionally, cases in GSE37418 were assigned as the external validation set 2 (*n* = 70).

**TABLE 1:** Summary of studied cohorts.

	Train set	Validation set 1	Validation set 2
GEO access	GSE85217	GSE85217	GSE37418
Microarray Platform	GPL22286	GPL22286	GPL570
Cases included (*n*)	371	160	70
Age (mean ± SD)	11.5 ± 9.2	11.7(±9.4)	8.4(±3.12)
**Overall survival**			
Year (mean ± SD)	5.1 ± 3.7	5.1(±4.1)	3.7(±1.8)
Status (alive/dead)	278/93	118/42	58/12
**Gender**			
Female	122	53	21
Male	179	104	49
**Histology**			
Classic	206	107	48
Des	38	16	5
LCA	43	15	15
MBEN	5	0	2
**Subgroup**			
SHH	84	37	10
WNT	43	19	8
GROUP3	64	27	16
GROUP4	180	77	34

*GEO, Gene Expression Omnibus; SD, standard deviation; Des, desmoplastic/nodular; LCA, large-cell anaplastic; MBEN, medulloblastoma with extensive nodularity; SHH, sonic hedgehog; WNT, Wingless.*

**FIGURE 1:**
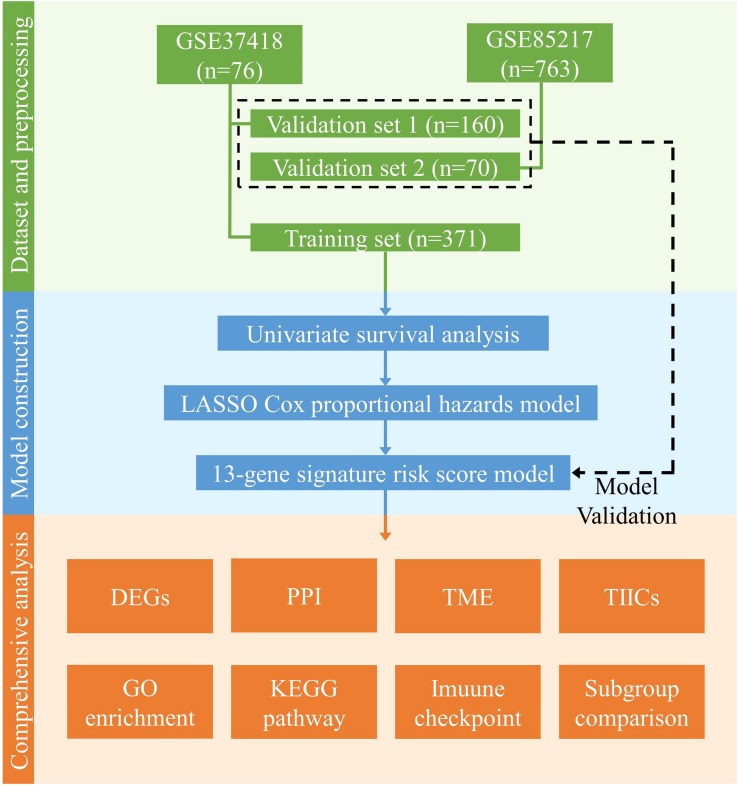
Design and workflow of this study. Public medulloblastoma datasets, including 763 (GSE85217) and 76 cases (GSE37418), were included in our analysis. Cases in GSE85217 were divided into the training set (*n* = 371) and validation set 1 (*n* = 160), and cases in GSE37418 were assigned as validation set 2 (*n* = 70) for cross-platform and external validation. We constructed our gene-signature-based risk score model using a training set and validated with two validation sets. Using the constructed model, we then performed comprehensive analysis, including analysis of DEGs, PPI network, TME, TIICs, immune checkpoint, KEGG pathways, and GO enrichment, as well as a comparison of established medulloblastoma molecular and histological subgroups.

The clinical characteristics of the datasets were summarized in Table 1, and the study design and workflow were summarized in Figure 1.

### Construction of the Gene Signature Model

**FIGURE 2:**
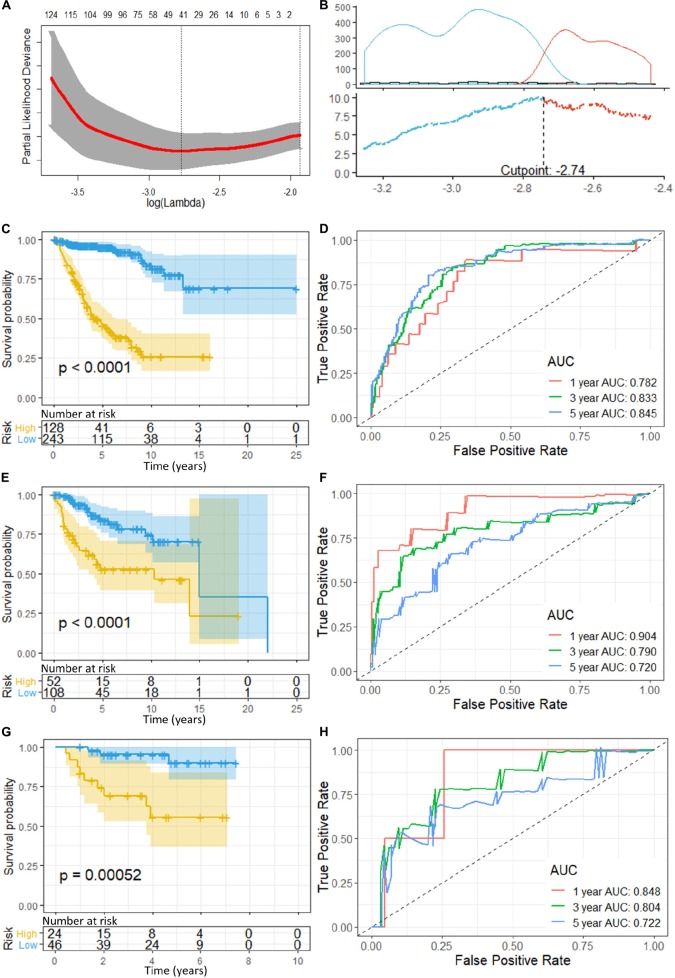
**(A)** Cross-validation of LASSO, the optimized log (lambda) was indicated by the dot line; **(B)** Optimizing the cutoff of the risk score using “maximally selected rank statistics method,” and an optimal cutoff of –2.74 was indicated; **(C)** K-M curve and **(D)** ROC of the training set; **(E)** K-M curve and **(F)** ROC of the validation set 1; and **(G)** K–M curve and **(H)** ROC of the validation set 2. *P*-value < 0.05 were considered statistically significant. Greater AUCs reflects better discrimination, while AUCs close to 0.5 reflects no discrimination. AUC, area under curve.

Prognostic model was constructed using the training set (*n* = 371, GSE85217). We included 21028 of 21641 probes with description from the manufacture and unambiguously mapped genes, from the microarray platform (GPL22286). A univariate survival analysis was performed, and we identified 2,438 OS-related genes (Supplementary Table 1) (*p* < 0.05). We further screened these genes using LASSO-penalized regression, and 14 genes with none-zero coefficient was selected by cross-validation (Figure 2A and Table 2). Among them, we excluded ENSG00000186838 (Selenoprotein V, *SELENOV*), which cannot be properly mapped in the GPL570 platform. Finally, a total of 13 genes were selected for the construction of prognostic model, including cytochrome B5 domain containing 2 (*CYB5D2*), filamin binding LIM protein 1 (*FBLIM1*), interleukin 27 receptor subunit alpha (*IL27RA*), cell migration inducing hyaluronidase 1 (*CEMIP*), dynein axonemal heavy chain 2 (*DNAH2*), pitrilysin metallopeptidase 1 (*PITRM1*), FKBP prolyl isomerase 4 (*FKBP4*), Cyclin Y (*CCNY*), phospholipase A2 Group IVE (*PLA2G4E*), immunoglobulin kappa variable 1/OR2-108 (*IGKV1OR2-108*), ZFP3 Zinc Finger Protein (*ZFP3*), XK related 5 (*XKR5*), and spectrin repeat containing nuclear envelope family member 3 (*SYNE3*). Most of the 13 signature genes (except for *IGKV1OR2-108*, *ZFP3*, *XKR5*, and *SYNE3*) were reported to be related to neurological functions and diseases, which are summarized in Table 3 and will be further discussed in detail.

**TABLE 2:** Gene signature selected for model construction.

Probe	Gene	Coefficient	Factor	Expression level (high vs. low risk)
ENSG00000167740	*CYB5D2*	–0.244845303	Protective	Decreased
ENSG00000188089	*PLA2G4E*	–0.2109008	Protective	Decreased
ENSG00000162458	*FBLIM1*	–0.199451582	Protective	Decreased
ENSG00000104998	*IL27RA*	–0.193108399	Protective	Decreased
ENSG00000186838*	*SELENOV*	–0.168074864	Protective	Decreased
ENSG00000231292	*IGKV1OR2-108*	–0.116666216	Protective	Decreased
ENSG00000183914	*DNAH2*	–0.057694631	Protective	Decreased
ENSG00000103888	*CEMIP*	–0.054565913	Protective	Decreased
ENSG00000180787	*ZFP3*	–0.021712839	Protective	Decreased
ENSG00000275591	*XKR5*	–0.014694104	Protective	Decreased
ENSG00000108100	*CCNY*	0.02622514	Risk	Increased
ENSG00000107959	*PITRM1*	0.104753688	Risk	Increased
ENSG00000176438	*SYNE3*	0.108748445	Risk	Increased
ENSG00000004478	*FKBP4*	0.196424875	Risk	Increased

*Excluded: no mapped gene in GLP570 platform. Gene names: Cytochrome B5 Domain Containing 2 (*CYB5D2*), Phospholipase A2 Group IVE (*PLA2G4E*), Filamin Binding LIM Protein 1 (*FBLIM1*), Interleukin 27 Receptor Subunit Alpha (*IL27RA*), Selenoprotein V (*SELENOV*), Immunoglobulin Kappa Variable 1/OR2-108 (*IGKV1OR2-108*), Dynein Axonemal Heavy Chain 2 (*DNAH2*), Cell Migration Inducing Hyaluronidase 1 (*CEMIP*), ZFP3 Zinc Finger Protein (*ZFP3*), XK Related 5 (*XKR5*), Cyclin Y (*CCNY*), Pitrilysin Metallopeptidase 1 (*PITRM1*), Spectrin Repeat Containing Nuclear Envelope Family Member 3 (*SYNE3*), and FKBP Prolyl Isomerase 4 (*FKBP4*).

**TABLE 3:** Summary of signature genes related to neurological functions or diseases.

Gene	Neurological functions and diseases	CNS tumors	Other tumors or tumor-related pathways
*CYB5D2*	Neurogenesis, neural functions		Tumorigenesis and cancer progression, breast and cervical cancer
*FBLIM1*	Brain development, autism spectrum disorders	Migration and invasion in glioma	
*IL27RA*	Immune regulation in CNS		
*CEMIP*	Brain function and development, Schwann cell dedifferentiation	Immune response in glioblastoma	Wingless (WNT) signaling
*DNAH2*	Parkinson’s disease, autism, adult-onset hearing loss		Clear cell renal cell carcinomas
*PITRM1*	Amyloidogenic neuropathy, Alzheimer’s disease		
*FKBP4*	Neuronal differentiation, chemotropic guidance of neuronal growth cones, regulating neuroprotective activities with calcium channels, major depressive disorder		Prostate-cancer
*CCNY*	Synapse formation, synapse elimination, hippocampal neurons related pathways, hippocampal long-term potentiation	Glioma cell proliferation	
*PLA2G4E*	Neurobehavioral disorders		

*Gene names: Cytochrome B5 Domain Containing 2 (*CYB5D2*), Filamin Binding LIM Protein 1 (*FBLIM1*), Interleukin 27 Receptor Subunit Alpha (*IL27RA*), Cell Migration Inducing Hyaluronidase 1 (*CEMIP*), Dynein Axonemal Heavy Chain 2 (*DNAH2*), Pitrilysin Metallopeptidase 1 (*PITRM1*), FKBP Prolyl Isomerase 4 (*FKBP4*), Cyclin Y (*CCNY*), and Phospholipase A2 Group IVE (*PLA2G4E*).*

Risk scores for each patient can be calculated as:

$$\begin{array}{l} \text{Risk}\;\text{Score}\\ =(-0.244845303*CYB5D2)+(-0.2109008*PLA2G4E)\\ \;+(-0.199451582*FBLIM1)+(-0.193108399*IL27RA)\\ \;+(-0.116666216*IGKV1OR2-108)\\ \;+(-0.057694631*DNAH2)+(-0.054565913*CEMIP)\\ \;+(-0.021712839*ZFP3)+(-0.014694104*XKR5)\\ \;+(0.02622514*CCNY)+(0.104753688*PITRM1)\\ \;+(0.108748445*SYNE3)+(0.196424875*FKBP4) \end{array}$$

“Maximally selected rank statistics method” indicated an optimal cutoff of −2.74 (Figure 2B) for the training set. Patients with a risk score of less than or equal −2.74 were classified into low-risk groups, while those above −2.74 were classified into high-risk groups. K–M curves were plotted, and patients in the high-risk group demonstrated significant worse OS than those in the low-risk group (high-risk *n* = 128, low-risk *n* = 243, *p* < 0.0001) (Figure 2C). ROC analysis was performed to assess the accuracy of the risk score model, and the AUCs for 1-, 3-, and 5-year OS were 0.782, 0.833, and 0.845, respectively (Figure 2D).

### Validation of the Gene Signature Model

To validate the gene signatures, we applied the constructed model to the validation set 1 (*n* = 160, GSE85217), resulting in 52 high-risk cases and 108 low-risk cases, respectively. K–M curves demonstrated a significantly lower OS of the high-risk group compared with that of the low-risk groups (high-risk *n* = 52, low-risk *n* = 108, *p* < 0.0001) (Figure 2E). The ROC yielded AUCs of 0.904, 0.790, and 0.720 for 1-, 3-, and 5- year OS prediction, respectively (Figure 2F). These analyses indicated a great performance of our 13-gene signature model predicting the OS of the patients in the validation set 1.

**FIGURE 3:**
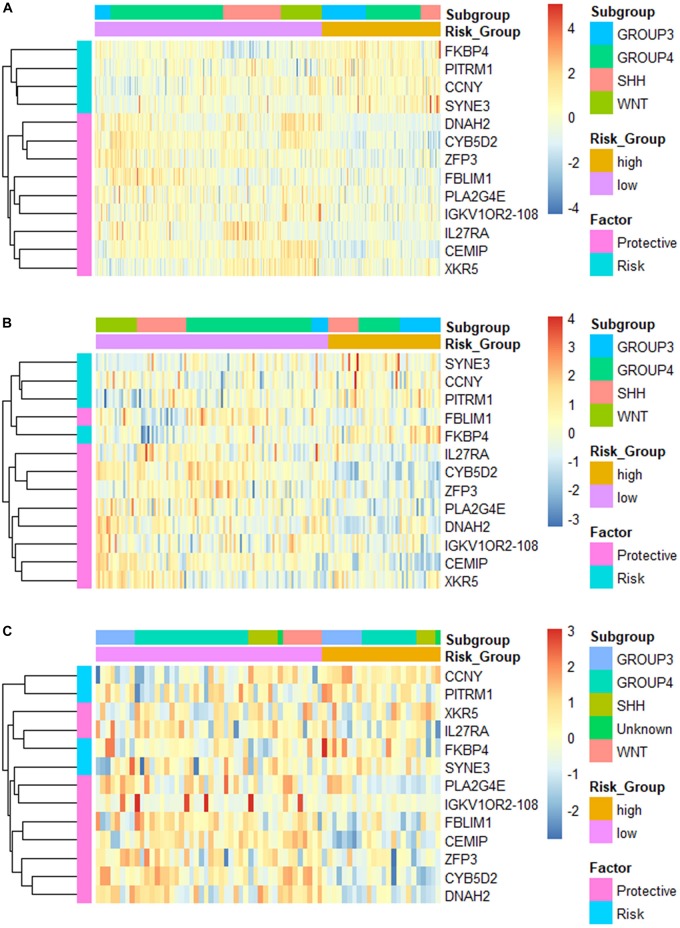
Heatmap of the constructed 13-gene signature for **(A)** training set; **(B)** validation set 1; and **(C)** validation set 2, clustered by gene expression level and risk groups.

Additionally, we applied the constructed model to validation set 2 (*n* = 70, GSE37418), which was generated with a different study group and microarray platform (GPL570), to further validate its applicability. In validation set 2, 24 patients were predicated to be in the high-risk group while 46 were predicated to be in the low-risk group. K–M curves demonstrated a significant lower OS of the high-risk group compared with low-risk group (high-risk *n* = 24, low-risk *n* = 46, *p* = 0.00052) (Figure 2G). The ROC yielded AUCs of 0.848, 0.804, and 0.722 for 1-, 3-, and 5- year OS prediction, respectively (Figure 2H). These results were very similar to our training and validation set 1, indicating a consistent performance of our model when applied to an external, cross-platform dataset. To visualize the performance of the gene signature, heatmaps were plotted for the 13-gene signature in each dataset (Figure 3).

### Independent Predictive Value of the Gene Signature

Patients in the validation set 1 (*n* = 160) and validation set 2 (*n* = 70) were used to assess the independent predictive value of the 13-gene signature. Univariant Cox analysis demonstrated that the OS for patients was significantly associated with risk groups in both validation set 1 (Hazard ratio, HR = 0.31, *p* = 0.00022) and validation set 2 (HR = 0.14, *p* = 0.003), while no significant correlation with clinical factors including age, gender, and molecular subgroups were observed (Table 4). The risk group was the only factor significantly associated with OS in the multivariant cox analysis (Validation set 1: HR = 0.3, *p* = 0.00107; Validation set 2: HR = 0.14, *p* = 0.00407). Although a significant association between histology types and OS was observed in the validation set 1 in univariate analysis (HR = 1.7, *p* = 0.044), it was not significant when assessed in multivariant cox analysis (HR = 1.51, *p* = 0.12). These findings indicate that the risk group was an independent predictor for the OS.

**TABLE 4:** Univariate and multivariate survival analysis for validation sets.

	Validation set 1			Validation set 2		
	HR	95% CI	*p*-value	HR	95% CI	*p*-value
**Univariate analysis**						
Age (3–6 vs. 6–9 vs. 9–12 vs. 12–15 vs. 15–18 vs. 18+)	1	0.86–1.2	0.75	0.76	0.42–1.4	0.37
Gender (male vs. female)	1.4	0.7–3	0.33	0.78	0.23–2.6	0.69
Histology (classic vs. Des vs. LCA vs. MBEN)	1.7	1–2.7	0.044*	1.4	0.81–2.5	0.23
Risk Group (low vs. high)	0.31	0.17–0.58	0.00022*	0.14	0.037–0.51	0.003*
Subgroup (SHH vs. WNT vs. Group 3 vs. Group 4)	1	0.8–1.4	0.73	0.8	0.5–1.3	0.35
**Multivariate analysis**						
Age (3–6 vs. 6–9 vs. 9–12 vs. 12–15 vs. 15–18 vs. 18+)	1.17	0.95–1.47	0.14	0.77	0.41–1.44	0.41
Gender (male vs. female)	1.08	0.51–2.30	0.84	0.86	0.20–3.64	0.84
Histology (classic vs. Des vs. LCA vs. MBEN)	1.51	0.89–2.52	0.12	1.48	0.78–2.78	0.23
Risk Group (low vs. high)	0.3	0.14–0.61	0.00107*	0.14	0.04–0.54	0.00407*
Subgroup (SHH vs. WNT vs. Group 3 vs. Group 4)	1.23	0.89–1.68	0.21	0.83	0.47–1.45	0.51

*HR, hazard ratio; CI, confidence interval; Des, desmoplastic/nodular; LCA, large-cell anaplastic; MBEN, medulloblastoma with extensive nodularity; SHH, sonic hedgehog; WNT, Wingless.*

### Comparison of Risk Scores Among Molecular and Histological Subgroups

**FIGURE 4:**
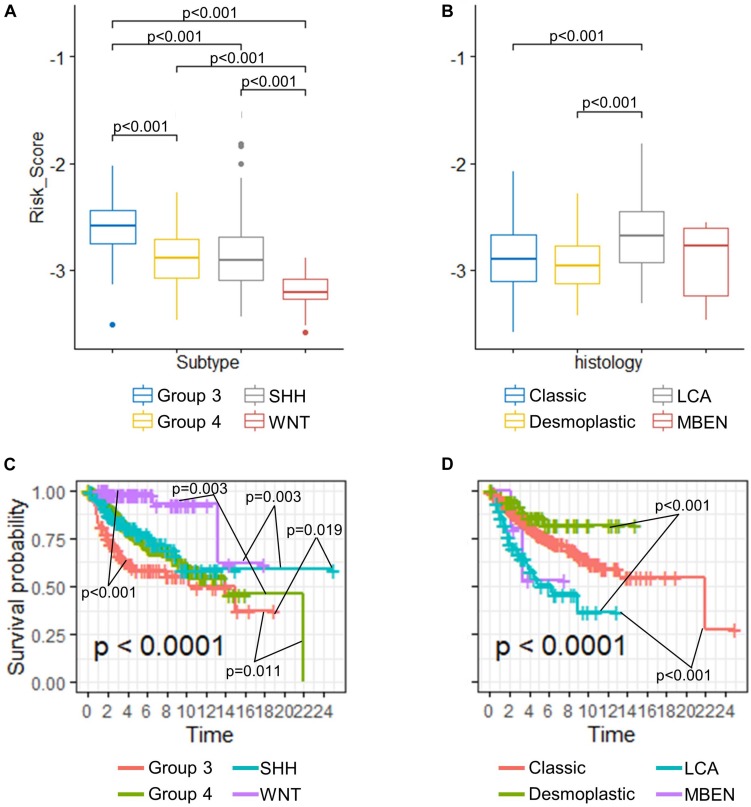
Comparison of risk scores in different **(A)** molecular subgroups and **(B)** histological subtypes (Kruskal–Wallis *H* test followed by Dunn’s *post hoc* tests for pairwise comparisons). K–M curves of different **(C)** molecular subgroups and **(D)** histological subtypes (*P*-values were calculated using “survminer” and were adjusted using the BH method).

Despite proven to be an independent predictor of the OS, the risk score seemed to vary among different medulloblastoma subgroups. We then compared the risk scores among established medulloblastoma molecular subgroups, including SHH, WNT, group 3, and group 4, as well as histology types, including classic, desmoplastic, LCA, and medulloblastoma with extensive nodularity (MBEN), using the GSE85217 dataset. We found that the risk score of each molecular and histological subgroup was consistent with its OS. For example, Group 3 medulloblastomas, known to feature the worst survival, were found to have the highest risk scores (Figure 4A) and the worst OS (Figure 4C), whereas WNT tumors, which are thought to have by far the best survival, demonstrated the lowest risk score (Figure 4A) and the best OS among all molecular subgroups (Figure 4C) (Kool et al., 2012; Taylor et al., 2012; Shih et al., 2014). LCA medulloblastomas were found to have the highest risk score, which was significantly higher than that of classic (*p* < 0.001) and desmoplastic medulloblastomas (*p* < 0.001) (Figure 4B). Consistently, LCA demonstrated the worst OS among all histology types, which was significantly shorter than that of classic (*p* < 0.001) and desmoplastic medulloblastomas (*p* < 0.001) (Figure 4D).

### Differentially Expressed Gene (DEG), GO Enrichment, and KEGG Analysis

**FIGURE 5:**
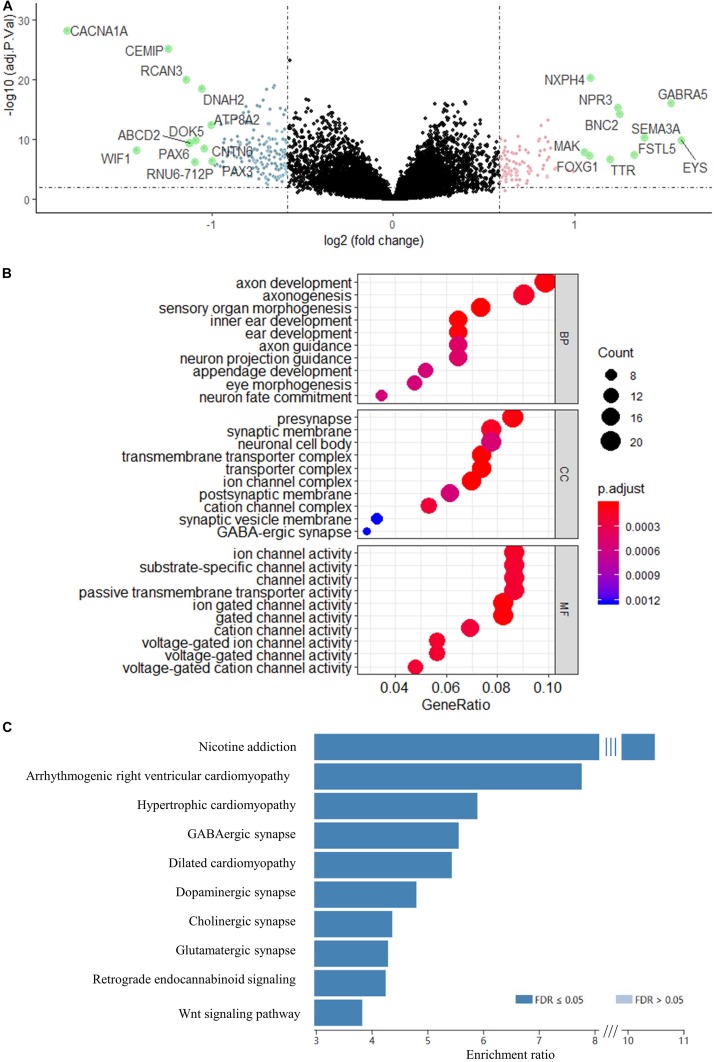
**(A)** Volcano map of DEGs between risk groups. Genes with a red color were upregulated, whereas genes with a blue were downregulated. Genes with absolute Log2FC > 1 are highlighted in green. **(B)** GO enrichment analysis of the DEGs. BP, biological process; CC, cellular component; MF, molecular function. **(C)** KEGG analysis of the DEGs. Genes were selected by “TOP” 10 method using WEB-based Gene Set Analysis Toolkit.

Using the validated gene signature model and expression data of GSE85217 (high-risk *n* = 180, low-risk *n* = 351), we identified 265 DEGs (Figure 5A and Supplementary Table 2) between the high-risk and low-risk group. GO analysis demonstrated that these genes were highly related to neurological functions, as DEGs were found to be significantly enriched in biological processes, such as axon development, axonogenesis, axon guidance, neuron projection guidance, and neuron fate commitment; cellular components, such as presynapse, synaptic membrane, neuronal cell body, postsynaptic membrane, synaptic vesicle membrane, and GABA-ergic synapse; and molecular functions, such as substrate-specific channel activity and channel activities, including the ion channel, gated channel, cation channel, and voltage-gated channel (Figure 5B). A KEGG analysis demonstrated enrichment of DEGs in synapse-related pathways (Figure 5C and Supplementary Table 3) and the WNT signaling pathway, which was widely reported in medulloblastomas (Xia et al., 2019; Majd and Penas-Prado, 2019).

### PPI Network Analysis and Prediction of Hub Genes and Network Modules

**FIGURE 6:**
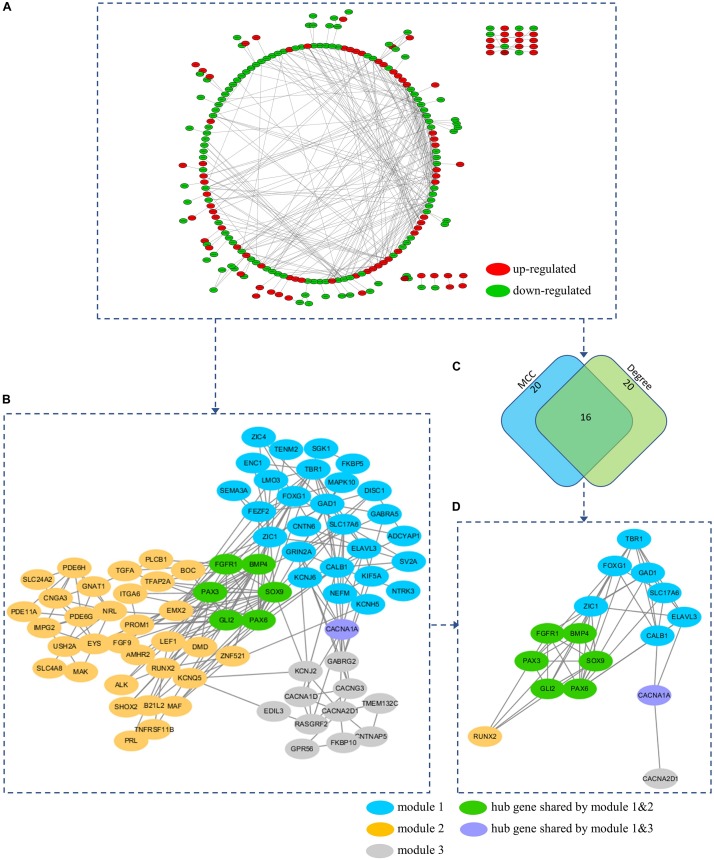
**(A)** PPI network of the DEGs. **(B)** A total of 3 modules (blue, yellow, and gray nodes) were identified from the PPI network using MCOD. **(C)** Hub genes were predicted based on the MCC and Degree method using Cytohubba. **(D)** A total of 16 hub genes were predicted from the PPI network. All hub genes can be found in at least on module. Hub gene *FGFR1*, *BMP4*, *PAX3*, *SOX9*, *GLI2*, and *PAX6* were presented in both module 1 and 2 (Green nodes). Hub gene *CACNA1A* was presented in both module 1 and 3 (Purple nodes).

To further understand the interaction between the DEGs and their roles in medulloblastoma, PPI network of these genes was constructed using STRING database and was visualized using Cytoscape. STRING analysis identified 254 nodes and 400 edges in this PPI network (enrichment *p* < 0.001) (Figure 6A and Supplementary Table 4). Using MCODE, three modules were identified from the PPI network (Figure 6B and Supplementary Table 6).

**FIGURE 7:**
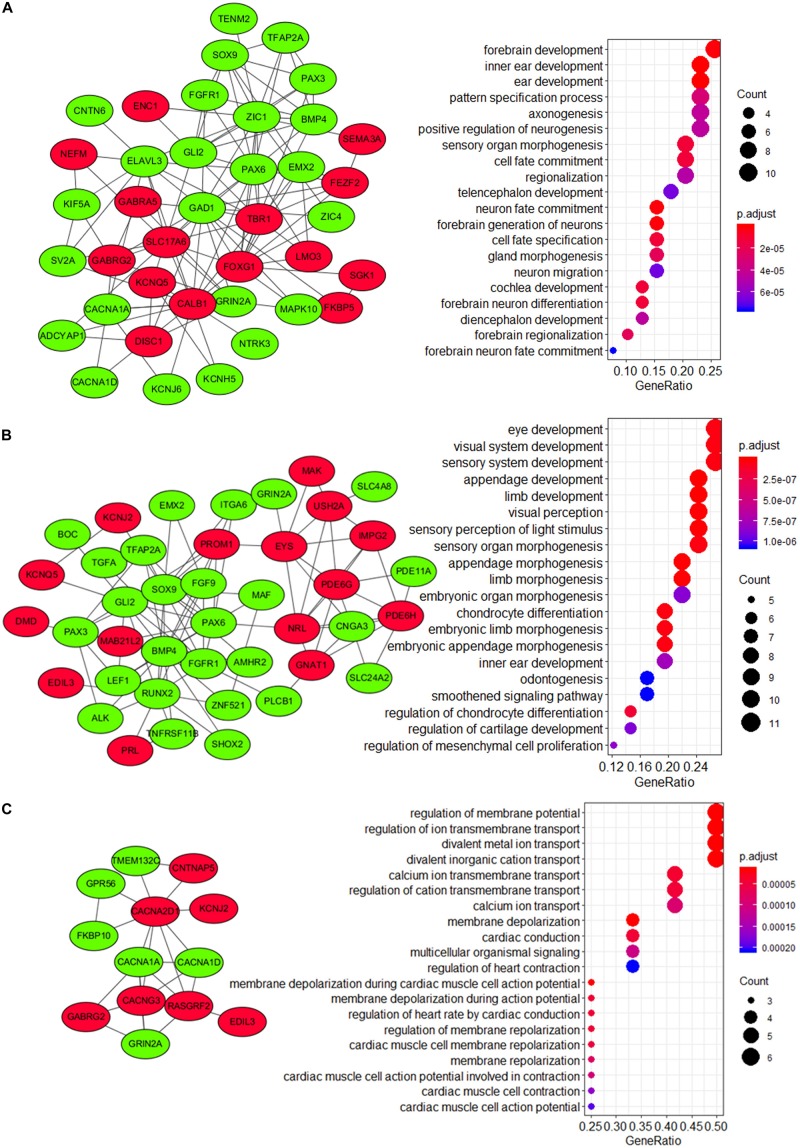
Visualization and GO enrichment analysis of DEGs in **(A)** module 1, **(B)** module 2, and **(C)** module 3. DEGs that were upregulated in the high-risk group are in red nodes, whereas those that were downregulated are in green nodes. **(A)** DEGs in module 1 were significantly enriched in pathways that are related to CNS development, while **(B)** DEGs in module 2 were significantly enriched in pathways that are related to embryonic development.

To explore the underlying mechanism of these modules, a GO enrichment analysis was performed for each module. Interestingly, we found that DEGs in module 1 were significantly enriched in pathways that were related to the development and function of CNS (including forebrain development, axonogenesis, positive regulation, telencephalon development, neuron fate commitment, forebrain generation of neurons, neuron migration, forebrain neuron differentiation, diencephalon development, forebrain regionalization, and forebrain neuron fate commitment) (Figure 7A and Supplementary Table 7), while those in module 2 were significantly enriched in pathways that were related to embryonic development (including eye development, visual system development, sensory system development, appendage development, limb development, inner ear development, visual perception, sensory perception of light stimulus, sensory organ morphogenesis, appendage morphogenesis, limb morphogenesis, embryonic organ morphogenesis, chondrocyte differentiation, embryonic limb morphogenesis, embryonic appendage morphogenesis, odontogenesis, regulation of chondrocyte differentiation, regulation of cartilage development, and regulation of mesenchymal cell proliferation) (Figure 7B and Supplementary Table 7). Therefore, module 1 and module 2 might be involved in the development of medulloblastomas, which are known as embryonal CNS tumors. For DEGs in module 3, however, the underlying mechanism and its relevance to medulloblastomas were less clear (Figure 7C). Notably, four subunits of the calcium voltage-gated channel (CACN), which has been reported as a novel target for medulloblastoma therapy (Phan et al., 2017; Huang et al., 2018), were predicted, including calcium voltage-gated channel subunit alpha1 A (*CACNA1A*), calcium voltage-gated channel subunit alpha1 D (*CACNA1D*), calcium voltage-gated channel auxiliary subunit alpha2delta 1 (*CACNA2D1*), and calcium voltage-gated channel auxiliary subunit gamma 3 (*CACNG3*).

Hub genes were highly interconnected with network nodes, and intramodular hub genes in disease related modules have been reported to have clinical significance (Ivliev et al., 2010; Langfelder et al., 2013). Using Cytohubba, a total of 16 hub genes were predicted, including fibroblast growth factor receptor 1 (*FGFR1*), GLI family zinc finger 2 (*GLI2*), glutamate decarboxylase 1 (*GAD1*), *CACNA1A*, paired box 3 (*PAX3*), paired box 6 (*PAX6*), T-Box brain transcription factor 1 (*TBR1*), bone morphogenetic protein 4 (*BMP4*), calbindin 1 (*CALB1*), solute carrier family 17 member 6 (*SLC17A6*), SRY-Box transcription factor 9 (*SOX9*), Forkhead box G1 (*FOXG1*), Zic Family Member 1 (*ZIC1*), ELAV like RNA binding protein 3 (*ELAVL3*), RUNX family transcription factor 2 (*RUNX2*), and *CACNA2D1* (Figures 6C,D and Supplementary Table 5). All 16 hub genes were presented in at least one of network modules predicted by MCOD. In addition, 6 hub genes were presented in both module 1 and 2 (*FGFR1*, *BMP4*, *PAX3*, *SOX9*, *GLI2*, and *PAX6*), while 1 hub gene was presented in both module 1 and 3 (*CACNA1A*) (Figures 6B,D). All hub genes presented in multiple modules have been reported in medulloblastomas which would be further discussed in the discussion section. These hub genes might be critical in the underlying mechanism of the PPI network.

### Association of TME With the Risk Score Model and Molecular Subgroups of Medulloblastoma

**FIGURE 8:**
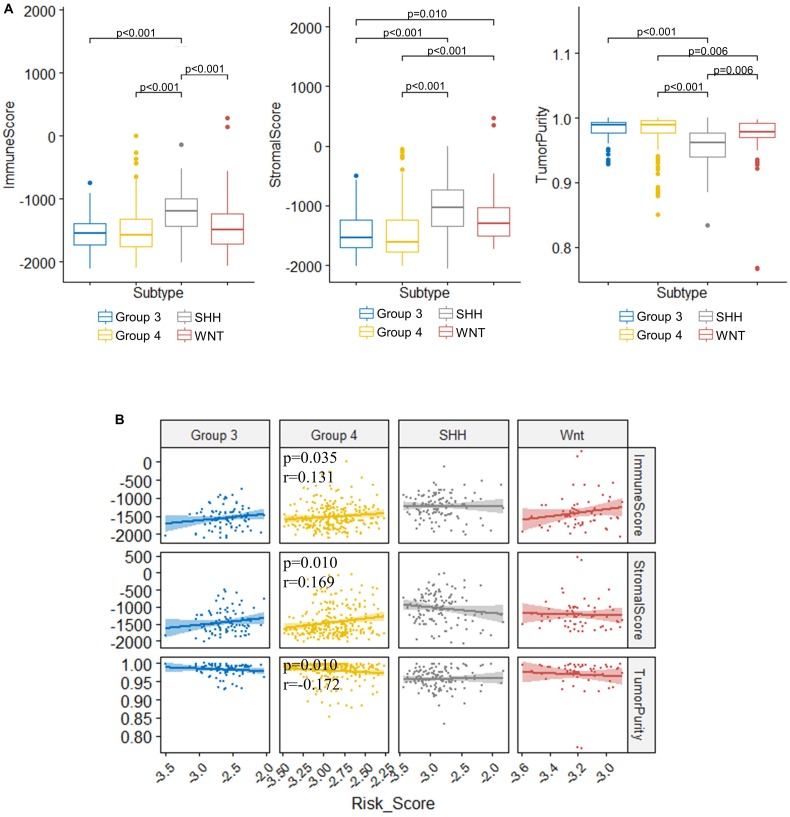
Significant correlations between the risk score and the immune score, stromal score, and tumor purity were found in the TME of Group 4 medulloblastomas. **(A)** Comparison of immune score, stromal score, and tumor purity between molecular subgroups (Kruskal–Wallis *H* test followed by Dunn’s *post hoc* tests for pairwise comparisons). **(B)** The correlation between risk scores and immune scores, stromal scores, as well as tumor purity (Spearman’s *r* and *p*. *P* values were adjusted using BH method. Only significant *p*-values were presented).

TME is essential for tumor development and is involved in the drug resistance in cancers (Quail and Joyce, 2017). To understand the role of our risk score model in the TME of medulloblastomas, we inferred the stromal and immune cell admixture, which are of important in TME, as well as the tumor purity in the GSE85217 dataset using ESTIMATE algorithm. We first compared the TME in different molecular subgroups (Figure 8A). SHH tumors had the highest immune and stromal score but the lowest tumor purity among all subgroups. WNT tumors had a significantly lower immune score (*p* < 0.001) and higher tumor purity (*p* = 0.006) than SHH tumors, a significantly higher stromal scores than Groups 3 (*p* = 0.010) and Group 4 (*p* < 0.001) tumors, as well as a significantly lower tumor purity than Group 4 tumors (*p* = 0.006). The TME of Group 3 and Group 4 medulloblastomas seemed to be similar, as no significant differences in terms of the immune score (*p* = 1.000), stromal score (*p* = 1.000), and tumor purity (*p* = 1.000) were detected between these groups. We then analyzed the correlation between TME and the risk score (Figure 8B). The risk score of Group 4 tumors were positively correlated with both the stromal score (*r* = 0.169, *p* = 0.010) and the immune score (*r* = 0.131, *p* = 0.035) while negatively correlated with the tumor purity (*r* = −0.172, *p* = 0.010) (Figure 8B). No significant correlations were detected in other subgroups. We also compared the TME between different risk groups classified by our risk model (Supplementary Table 8), and found that high-risk group had significantly lower tumor purity compared to those classified into low-risk group (*p* = 0.045) in Group 4 medulloblastomas, while high-risk Group 3 medulloblastomas demonstrated significantly higher level of stromal scores compared to those classified as low-risk group in the same subgroup (*p* = 0.045). These findings might be suggestive of a subgroup-specific TME in medulloblastomas and indicated an association between our risk score model and these distinct TME profiles in Group 4 and SHH medulloblastoma.

### Association of TIICs and Immune Checkpoint With the Risk Score Model

**FIGURE 9:**
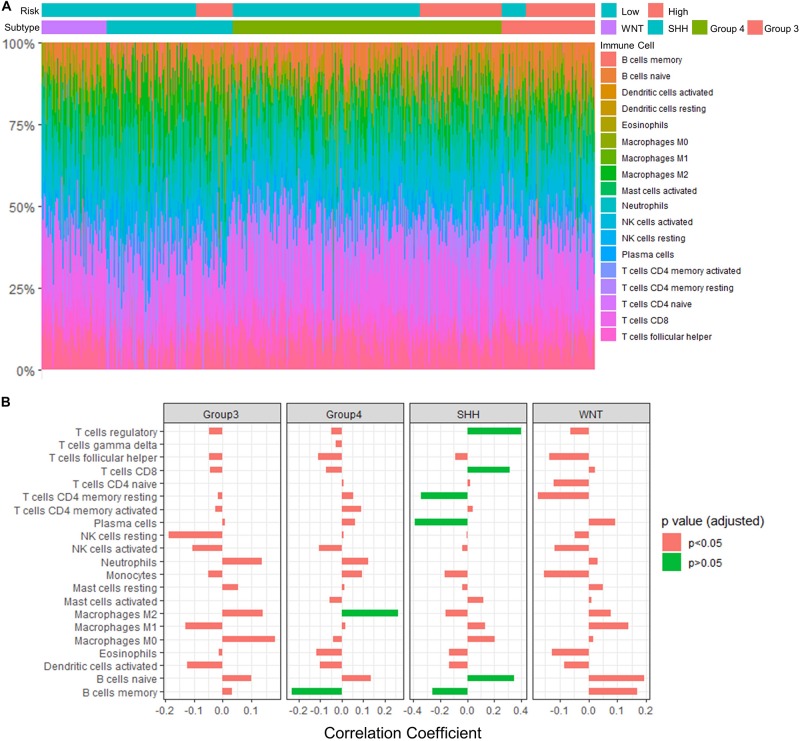
Significant correlations between the risk score and the fraction of TIICs were found in Group 4 and SHH medulloblastomas. **(A)** TIICs profile in GSE85217 dataset estimated using CIBERSORTx algorithm. **(B)** The correlation between the risk score and the fraction of infiltrating immune cells (Spearman’s *r* and *p*. *P*-values were adjusted using BH method). Significant correlations are in green color.

To assess the relation between immune cell infiltration and our 13-gene signature, we profiled 22 TIICs using CIBERSORTx algorithm in the GSE85217 dataset (Figure 9A and Supplementary Table 9). We first investigated the correlation between the risk score and the immune cells (Figure 9B and Supplementary Table 10). For SHH cases, the risk score was positively correlated with naïve B cells (*r* = 0.347, *p* < 0.001) but negatively correlated with memory B cells (*r* = −0.259, *p* = 0.013) and plasma cells (*r* = −0.388, *p* < 0.001). In addition, the risk score of SHH cases was positively correlated with CD8 T cells (*r* = 0.316, *p* = 0.002) and regulatory T cells (*r* = 0.400, *p* < 0.001) but was negatively correlated with CD4 T cells (*r* = −0.343, *p* < 0.001). For Group 4 medulloblastomas, the risk score was negatively correlated with memory B cells (*r* = −0.232, *p* = 0.002) as well and positively correlated with M2 Macrophages (*r* = 0.262, *p* < 0.001).

We also examined the association between our risk score model and immune checkpoint pathways, focusing on *PD-L1* and cytotoxic T-lymphocyte associated protein 4 (*CTLA4*) since inhibitors targeting these checkpoints have been proposed to be effective in treating medulloblastoma animal models (Pham et al., 2016). We found that the risk score was significantly correlated with *PD-L1* expression (*p* < 0.001, *r* = −0.162) but not with *CTLA4* expression (Supplementary Table 11).

### Correlation of TIICs With the Signature Genes and PPI Network Hub Genes

**FIGURE 10:**
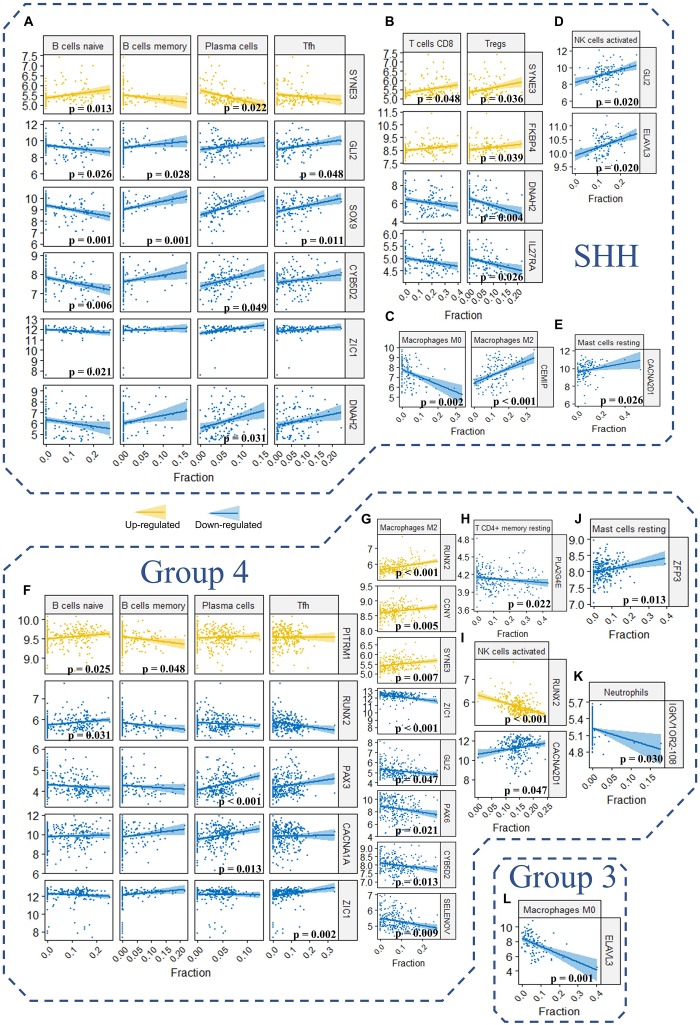
**(A–L)** The correlation between the expression level of the signature genes as well as hub genes and the fraction of the 22 TIICs in GSE85217 dataset (Spearman’s *r* and *p*. *P*-values were adjusted using BH method. Only genes and TIICs with significant correlations are presented).

To further understand the role of our signature genes and PPI network hub genes in immune cells infiltration, we assessed the correlation between signature genes as well as network hub genes and fraction of immune cells (Figure 10 and Supplementary Table 12). For the B cells in SHH tumors, naïve B cells were significantly correlated with *SOX9* (*p* = 0.001, *r* = −0.391), *GLI2* (*p* = 0.026, *r* = −0.303), *ZIC1* (*p* = 0.021, *r* = −0.315), *CYB502* (*p* = 0.007, *r* = −0.350), and *SYNE3* (*p* = 0.013, *r* = 0.331) expression (Figure 10A). Memory B cells were significantly correlated with *SOX9* (*p* = 0.001, *r* = 0.398) and *GLI2* (*p* = 0.028, *r* = 0.300) expression, and plasma cells were positively correlated with *CYB502* (*p* = 0.049, *r* = 0.279), *DNAH2* (*p* = 0.031, *r* = 0.296) and *SYNE3* (*p* = 0.048, *r* = 0.280) expression (Figure 10A). Considering that *SOX9*, *GLI2*, *ZIC1*, *CYB502*, and *DNAH2* were downregulated, whereas *SYNE3* was upregulated in the high-risk group, the expression pattern of these genes was in accordance with our aforementioned observation that the risk score was positively correlated with naïve B cells but negatively correlated with memory B cells and plasma cells in SHH tumors. Interestingly, *GLI2* and *SOX9* were also significantly correlated with follicular helper T cells (Tfh) in SHH tumors (*p* = 0.048, *r* = 0.280; *p* = 0.011, *r* = 0.337, respectively) (Figure 10A). Since Tfh is known to be essential in directing B cells differentiation into memory B cells and plasma cells in the germinal center, these genes might be important in the regulation of tumor-infiltrating B cells in SHH medulloblastomas.

Regarding other T cells in SHH medulloblastomas, CD8 T cells were significantly correlated with *SYNE3* expression (*p* = 0.048, *r* = 0.280), and regulatory T cells were significantly correlated with *SYNE3* (*p* = 0.036, *r* = 0.291), *FKBP4* (*p* = 0.039, *r* = 0.288), *IL27RA* (*p* = 0.026, *r* = −0.304), and *DNAH2* (*p* = 0.004, *r* = −0.362) expression (Figure 10B). Considering that *FKBP4* and *SYNE3* were upregulated, whereas *IL27RA* and *DNAH2* were downregulated in the high-risk group, the expression pattern of these genes was in accordance with our previous observation that the risk score was positively correlated with CD8 T cells and regulatory T cells. Regarding macrophages in SHH medulloblastomas, *CEMIP* expression was negatively correlated M0 (*p* = 0.002, *r* = −0.378) while positively correlated with M2 macrophages (*p* < 0.001, *r* = 0.437) (Figure 10C). Since *CEMIP* was downregulated in the high-risk group, this finding was consistent with our observation of significantly increased fraction of M0 macrophages in the high-risk group. Moreover, activated NK cells were positively correlated with *GLI2* (*p* = 0.020, *r* = 0.318) and *ELAVL3* (*p* = 0.020, *r* = 0.317) expression (Figure 10D), and resting mastocyte cells were significantly correlated with *CACNA2D1* expression (*p* = 0.026, *r* = 0.303) in SHH medulloblastomas (Figure 10E).

For Group 4 medulloblastomas, B naïve cells were positively correlated with *RUNX2* (*p* = 0.031, *r* = 0.208) and *PITRM1* (*p* = 0.025, *r* = 0.214) expression, and the latter was negatively correlated with memory B cells as well (*p* = 0.048, *r* = −0.198) (Figure 10F). Plasma cells were positively correlated with both *PAX3* (*p* < 0.001, *r* = 0.360) and *CACNA1A* (*p* = 0.013, *r* = 0.228) expression (Figure 10F). These results were consistent with our observation that the risk score was negatively correlated with memory B cells. However, while the expression of both *PITRM1* (*p* = 0.025, *r* = 0.214) and *RUNX2* (*p* = 0.031, *r* = 0.208) was positively correlated with the fraction of naïve B cells, the former was upregulated whereas the latter was downregulated in the high-risk group (Figure 10F). This might explain non-significant correlation detected between risk score and naïve B cells in Group 4 tumors. Regarding T cells, Tfh was significantly correlated with *ZIC1* expression (*p* = 0.002, *r* = 0.266), and resting memory T CD4 cells was significantly correlated with *PLA2G4E* expression (*p* = 0.022, *r* = −0.217) (Figures 10F,H).

In concordance with the aforementioned observations, a positive correlation between M2 macrophages and the risk score was detected in Group 4 medulloblastoma. Consequently, we found that M2 macrophages fraction were positively correlated with the expression of *RUNX2* (*p* < 0.001, *r* = 0.326), *CCNY* (*p* = 0.005, *r* = 0.252) and *SYNE3* (*p* = 0.007, *r* = 0.243) expression, which were upregulated in the high-risk group, while negatively correlated with the expression of *ZIC1* (*p* < 0.001, *r* = −0.307), *GLI2* (*p* = 0.047, *r* = −0.200), *PAX6* (*p* = 0.021, *r* = −0.219), *CYB5D2* (*p* = 0.013, *r* = −0.230), and *SELENOV* (*p* = 0.009, *r* = −0.239), which were downregulated in the high-risk group (Figure 10G). Activated NK cells were correlated with *CACNA2D1* (*p* = 0.047, *r* = 0.199) and *RUNX2* (*p* < 0.001, *r* = −0.325) expression (Figure 10I). Resting mastocyte cells were correlated with *ZFP3* expression (*p* = 0.013, *r* = 0.231) (Figure 10J). Neutrophils were correlated with *IGKV1OR2-108* expression (*p* = 0.030, *r* = −0.210) (Figure 10K). For Group 3 tumors, M0 Macrophages were significantly correlated with *ELAVL3* expression (*p* = 0.001, *r* = −0.470) (Figure 10L).

Taken together, these findings indicated that our risk score model might be involved in the TIICs of medulloblastomas. The signatures genes and PPI network hub genes significantly correlated with immune cells may play a critical role in the medulloblastoma immune microenvironment, and their clinical significance requires further investigation.

## Discussion

Previous studies have improved our understanding of medulloblastoma dramatically, but a robust prognostic signature has yet to be established for medulloblastoma patients. The risk stratification for medulloblastoma was suggested long ago without incorporating genetic findings (Packer et al., 2003). Magnetic resonance (MR) imaging-based signatures were promising in predicting molecular subgroups (Colafati et al., 2018) but have not been used in predicting patient prognosis. Although gene signatures were investigated in several studies, a major focus was on patient preselection for targeted therapy (Shou et al., 2015) or molecular classification (Corno et al., 2012; Chen et al., 2013). Northcott et al. (2012) presented a 22-subtype-specific gene signature that can predict molecular subgroup in 88% of recent Formalin Fixed Paraffin Embedded (FFPE) medulloblastoma samples (Northcott et al., 2012). Braoudaki et al. (2014) used a microRNA (miRNA) signature to predict the clinical outcome in pediatric CNS tumors, but only 34 medulloblastomas were analyzed with other brain tumors, identifying only two signature miRNAs, which were not used to construct predictive models. Tantawy et al. (2018) also investigated miRNA signature for medulloblastoma; however, only a total of 82 miRNAs were assessed, in a cohort of 30 medulloblastoma cases with another 90 pediatric brain tumors, resulting in only 1 miRNA that was specific to medulloblastoma. The prognostic model established by Tamayo et al. (2011) was the only model we found, that could be used to predict clinical outcomes based on expression profiles. While their model can predict tumor relapse, it was not constructed to predict survival of the patients.

In this study, we constructed a 13-gene signature risk score model predicting the OS of medulloblastoma patient. The robustness and applicability of this model was validated with two independent datasets, generated from two different microarray platforms. Our results demonstrated that this model can identify high-risk patients that have significantly shorter OS compared with low-risk patients. We further confirmed the 13-gene signature as an independent predictor when a variety of clinical factors were considered simultaneously. To our knowledge, this is the first study constructing and validating a gene signature-based prognostic model for medulloblastoma.

Most of the 13 signature genes were related to neurological functions and diseases, as well as CNS tumors and other tumors. *CYB5D2* was related to neurogenesis, neural functions, tumorigenesis, and cancer progression (Kimura et al., 2010; Ryu et al., 2017). It was reported in breast (Ojo et al., 2019) and cervical cancer as well (Xie et al., 2011, 2016a,b; Bruce and Rybak, 2014). *FBLIM1* was reported to promote migration and invasion in glioma (Ou et al., 2017), and participate in brain development and autism spectrum disorders (Kang et al., 2011; Pinto et al., 2014; Ishizuka et al., 2018). *IL27RA* might participate in immune regulation in CNS (Iwasaki et al., 2015; Yoshida and Hunter, 2015). *CEMIP* may be related to brain function and development (Yoshino et al., 2017, 2018), MEK/ERK-induced Schwann-cell dedifferentiation (Boerboom et al., 2017), immune response in glioblastoma (Motaln et al., 2012), and WNT signaling (Li et al., 2017; Duong et al., 2018; Liang et al., 2018). It was frequently reported in colorectal cancers as well (Fink et al., 2015; Zhang et al., 2017). *DNAH2* mutation was reported in Parkinson’s disease (Gaare et al., 2018), autism (Butler et al., 2015), adult-onset hearing loss (Lewis et al., 2018), and clear cell renal cell carcinomas (Arai et al., 2015). *PITRM1* was associated with amyloidogenic neuropathy (Boczonadi and Horvath, 2016; Brunetti et al., 2016; Smith-Carpenter and Alper, 2018) and Alzheimer’s disease (Alikhani et al., 2011; Deters et al., 2017). *FKBP4* was found to be associated with major depressive disorder (Binder et al., 2004; Tatro et al., 2009a, b) and might be critical to early steps in neuronal differentiation (Quintá and Galigniana, 2012), chemotropic guidance of neuronal growth cones (Shim et al., 2009), and regulating neuroprotective activities with calcium channels (Ruan et al., 2008). It was also reported in malignancies like prostate cancer (Bhowal et al., 2017; Joshi et al., 2017) and breast cancer (Ostrow et al., 2009). *CCNY* knockout can inhibit glioma cell proliferation (Xu et al., 2010). It was known to regulate synapse formation, synapse elimination (Ou et al., 2010; Park et al., 2011), hippocampal neurons related pathways, and hippocampal long-term potentiation (Cho et al., 2015; Joe et al., 2017). *PLA2G4E* was reported to be strongly expressed in the brain, especially in neurons (Ogura et al., 2016), and was associated with neurobehavioral disorders (Everson et al., 2019; Morimoto et al., 2018).

To better understand the molecular mechanism underlying the 13-gene signature, we identified 265 DEGs by comparing the high-risk and low-risk group and performed GO and KEGG enrichment analysis for these genes. The results demonstrated that the DEGs were significantly associated with axon formation, synapse components, neuron components, and cell channel activities. Medulloblastoma is thought to arise from the granule cell precursors during the cerebellar development (Oliver et al., 2005; Roussel and Hatten, 2011). Cerebellar development occurs in multiple regions, including the ventricular zone surrounding the fourth ventricle and the upper rhombic lip (Martirosian et al., 2016). The former generates GABAergic neurons, while the latter generates glutamatergic neurons of cerebellum (Butts et al., 2014). Interestingly, we found that both the GABAergic and glutamatergic synapse pathways were associated with the 265 DEGs identified using the risk score model. Also, a GO analysis revealed that these DEGs were significantly enriched in pathways related to axon development and functions, including axonogenesis and axon guidance. Axon guidance pathways, such as Eph/ephrin signaling, were reported to play important roles in malignant brain tumor (Nakada et al., 2004; Pasquale, 2008; Miao et al., 2009). The Eph/ephrin signaling system plays a key role in the invasion of medulloblastoma, and EPH Receptor B2, *EphB2*, was found to be critical for invasion of pediatric medulloblastoma (Sikkema et al., 2012). Additionally, our results are consistent with previous report, which identified DEGs related to axon guidance in medulloblastoma spheres and core versus migrating cells and suggested a novel potential role for axon guidance signaling in medulloblastoma-propagating cells (Morrison et al., 2013). Moreover, KEGG analysis revealed that the DEGs were significantly enriched in WNT signaling pathways, which is known to be strongly related to medulloblastomas (Louis et al., 2016; Majd and Penas-Prado, 2019; Xia et al., 2019). Together, our study suggested that the 13 signature genes may play critical roles in the development of medulloblastoma by regulating these enriched pathways and functions through the DEGs.

Furthermore, we analyzed the PPI network of the 265 DEGs and identified 3 modules. Interestingly, GO enrichment analysis revealed that module 1 and module 2 were highly enriched in pathways that were related to CNS development and embryonic development, respectively. Defined as an embryonal CNS tumor, medulloblastoma is thought to arise from disruptions during the development of cerebellum as a result of dysregulated genes and pathways, including the Notch, WNT/β-Catenin, Transforming growth factor-beta (TGF-β)/bone morphogenetic protein (BMP), SHH/Patched, and Hippo pathways (Roussel and Hatten, 2011) in embryonic development. The aberrantly regulated DEGs in modules 1 and module 2 might be involved in this process considering their functional characteristics. Additionally, these modules were interconnected via several hub genes, namely, *FGFR1*, *BMP4*, *PAX6*, *PAX3*, *SOX9*, and *GLI2*, all of which have been related to medulloblastomas in previous studies. Fibroblast growth factor receptor (FGFR) signaling is known to drive SHH medulloblastomas and is critical in regulating medulloblastoma invasion, and *FGFR1* has been demonstrated to mediate inhibition of SHH medulloblastoma growth (Kumar et al., 2018; Neve et al., 2019). Bone morphogenetic proteins (BMPs) are known to regulate SHH-induced granule cell progenitor proliferation during cerebellar development and cell migration and invasion in Group 4 medulloblastoma model (Merve et al., 2014). *BMP4* can inhibit medulloblastoma proliferation and induce differentiation of medulloblastoma cells (Grimmer and Weiss, 2008; Zhao et al., 2008). The Paired box (PAX) gene family play a critical role in embryonic development especially the development of CNS. *PAX6* participates neuronal fate determination and can be regulated by SHH signaling in medulloblastoma (Shahi et al., 2010). *PAX3* is known to be involved in tumors originated from neural crest and has been related to neural cell adhesion molecules polysialylation, and, subsequently, cell–cell and cell–substratum interactions in medulloblastoma cells (Mayanil et al., 2000; Wang et al., 2008). *SOX9* play an important role in glial fate determination and are commonly overexpressed in WNT and SHH medulloblastoma (Larsimont et al., 2015). *SOX9* has been reported as a critical transcription factor in MYCN Proto-Oncogene, BHLH Transcription Factor, *MYCN*-driven medulloblastoma (Swartling et al., 2014) and can be related to drug resistance of these tumors (Rahmanto et al., 2016). Interestingly, *SOX9* might function upstream of *GLI2*, which is known to be a major effector in the Hedgehog signaling, and act as a key driver of SHH medulloblastomas (Bar et al., 2007; Raleigh et al., 2018; Yin et al., 2019). These modules and genes are likely to play a important role in medulloblastomas and shed light on potential pathways and mechanism underlying our 13-gene signature model.

TME plays a critical role in the development, progression and treatment of brain tumors (Quail and Joyce, 2017). The risk score predicted by our 13-gene signature model was significantly correlated with the immune score, stromal score, and tumor purity in Group 4 medulloblastoma, indicating a potential role of our model in the TME. Furthermore, the prognostic significance of TIICs in the immune microenvironment of brain tumors has been investigated extensively, and immunotherapy has been proposed for medulloblastoma (Sonabend et al., 2012; Boussiotis and Charest, 2017). Bockmayr et al. (2018) suggested a subgroup-specific TIICs in medulloblastoma with distinct types of immunosuppression associated with macrophages and regulatory T cells or cytokines and immune checkpoints. Consistently, distinctive association between our model and molecular subgroups were identified, as the risk score was significantly related to naïve B cells, memory B cells, plasma cells, resting memory CD4 T cells, CD8 T cells, and regulatory T cells in SHH tumors, while it was significantly correlated with memory B cells and M2 macrophages in Group 4 tumors. Interestingly, we found that SHH medulloblastoma with higher risk score tend to have an increased fraction of naïve B cells but a decreased fraction of memory B cells and plasma B cells, suggesting a potential defection of naïve B cell activation or the subsequent maturation into plasma cells and memory B cells in the immune microenvironment of SHH medulloblastomas with a higher risk score. Moreover, we found that *GLI2* and *SOX9* might contribute to this disruption. Downregulation of these genes in cases with higher risk scores was not only associated with increased naïve B cells and decreased memory B cells and plasma cells but also a decreased fraction of Tfh cells, which is known to provide key signals to the B cells for their differentiation into plasma cells and memory B cells (McHeyzer-Williams et al., 2009). In summary, our 13-gene signature might be associated with the infiltrating B cells in the TME of SHH medulloblastoma. The underlying mechanism of this association and its clinical significance in medulloblastomas require further investigation.

Comprising up to ∼30% of the tumor mass, tumor-associated macrophages (TAMs) are the major component of the TME in brain tumors. Despite their function of promoting specific immunity, the presences of macrophages in TME are thought to be pro-tumorigenic and has been associated with tumor progression, immune evasion, and immune suppression (Hambardzumyan et al., 2016). In medulloblastoma, however, TAMs can improve patient outcome (Maximov et al., 2019). The paradoxical role of TAMs might be partially related to their different polarization which can be tumor killing (M1) or tumor promoting (M2) (Sica et al., 2008; Mills, 2012). Interestingly, the fraction of M2 macrophages in Group 4 medulloblastoma were positively correlated with the risk score and were significantly correlated with the expression level of four signature genes (*CCNY*, *SYNE3*, *CYB5D2*, and *SELENOV*) and four hub genes (*GLI2*, *PAX6*, *RUNX2*, and *ZIC1*). These findings may suggest our 13-gene signature model might participate the regulation of TAMs in the TME of Group 4 medulloblastomas.

The programmed death (*PD-1*) pathway is a promising therapeutic target for brain tumors (Topalian et al., 2016). However, although *PD-L1* blockage treatment can improve the survival of Group 3 medulloblastoma in animal models (Pham et al., 2016), several studies indicated an absence of *PD-L1* expression in medulloblastomas and suggested a limited value of immunotherapy with *PD1/PD-L1* inhibitor (Majzner et al., 2017; Vermeulen et al., 2017), while others suggested that *PD-L1* expression in medulloblastomas might be associated with infiltrating CD8+ T cells, and relatively high *PD-L1* expression can be seen in some SHH and WNT cases (Bockmayr et al., 2018; Murata et al., 2018). Our analysis indicated that the risk score calculated using our 13-gene signature model was significantly correlated with *PD-L1* expression and prognosis in medulloblastoma patients. Therefore, our risk model might have potential value in selecting candidate patients who may benefit from *PD-L1* clinical trials.

This study is subject to several limitations. Infant medulloblastoma were not included in the analysis due to their distinct genomic and clinical features. Inclusion of these tumors could bias the patient outcome, especially due to their enrichment in SHH and Group 3 subgroups (Ramaswamy et al., 2016a). In addition, since we aimed to identify robust signature genes that are closely related to the prognosis of medulloblastoma patient, we focused on probes that unambiguously mapped to protein-coding genes or genes with known functions, and we filtered out a very small proportion of probes (*n* = 613, 2.8% of all 21641 probes) that have no description from the manufacturer of microarray platforms. It is not implausible that some genes were excluded simply due to their limited studies at the time. Moreover, we used a conventional approach of selecting the representative probe for genes detected by multiple probe sets for genes without recommended match per manufacturer’s instruction by selecting the probe covers the targeted gene with the highest expression level. This approach may be improved by alternative methods such as scoring systems, which have claimed to have optimized mapping (Li et al., 2011); an even more accurate prediction may thus have been achieved. Finally, SHH-activated Tumor Protein P53 (*TP53)*-mutant medulloblastomas were not classified in this study. Although this subgroup was included in the resent WHO classification (Louis et al., 2016), the datasets used in this study, as well as most of the existing medulloblastoma datasets, does not provide this information or only provide for a small proportion of the cases. Further sequencing of these tumors with *p53* mutation might be needed so that the model could be revalidated with the most updated WHO classification.

In summary, using gene expression data from GEO, we constructed and validated a 13-gene signature risk score model predicting the overall survival of medulloblastoma patients that can effectively classify low-risk and high-risk groups. Most of these 13 signature genes were involved in neurological activities or disease and may play critical roles in medulloblastoma through DEGs that were found to be significantly enriched in pathways related to neurological functions. PPI analysis revealed gene modules highly related to CNS and embryonic development. The risk score was found to be associated with the TME and TIICs particularly in SHH and Group 4 medulloblastomas and was significantly correlated with *PD-L1* expression. The signature genes and PPI network hub genes might play a role in regulation of TIICs. Our study may complement the current WHO classification for prognosis prediction and clinical management of medulloblastoma patients. This study also has the potential to provide insight into the tumorigenesis and pathogenesis of medulloblastoma and provide candidate molecular targets for therapeutic studies in the future.

## Data Availability Statement

Publicly available datasets were analyzed in this study. This data can be found here: https://www.ncbi.nlm.nih.gov/geo/query/acc.cgi?acc=GSE85217; https://www.ncbi.nlm.nih.gov/geo/query/acc.cgi?acc=GSE37418.

## Author Contributions

CL, HZ, ZX, SW, and XL carried out the study design. CL and SW performed the patient cohort and data preprocessing. CL, HZ, and ZX performed the model construction and validation. CL, ZX, and YX performed the differentially expressed gene analysis. CL, HZ, and ZX carried out the protein–protein interaction analysis, tumor microenvironment analysis, and tumor-infiltrating immune cells analysis. CL and HZ performed the statistical analysis. CL was responsible for the manuscript preparation. DM, SW, and XL carried out the critical revision of the manuscript. XL was responsible for the study overview and coordination.

## Conflict of Interest

The authors declare that the research was conducted in the absence of any commercial or financial relationships that could be construed as a potential conflict of interest.

## Footnotes

1 http://www.ncbi.nlm.nih.gov/geo/

2 https://www.ncbi.nlm.nih.gov/geo/query/acc.cgi?acc=GPL22286

3 https://www.ncbi.nlm.nih.gov/geo/query/acc.cgi?acc=GPL570

4 http://www.webgestalt.org

5 http://string-db.org

6 cibersortx.stanford.edu
